# Bridging UTAUT and HBM: determinants of wearable device adoption among chronic disease patients

**DOI:** 10.3389/fpubh.2025.1687887

**Published:** 2026-01-27

**Authors:** Zhaoxia Guo, Shih-Chih Chen

**Affiliations:** 1 School of Physical Education and Health Engineering, Taiyuan University of Technology, Taiyuan, Shanxi, China; 2 Department of Information Management, National Kaohsiung University of Science and Technology, Kaohsiung, Taiwan

**Keywords:** chronic disease patients, perceived severity, perceived susceptibility, performance expectancy, physical activity, wearable devices

## Abstract

**Objectives:**

Chronic diseases have emerged as a significant global health threat, making the effective management of these conditions crucial for improving patients’ quality of life. Wearable devices, a significant innovation in digital healthcare, offer new solutions for managing the health of patients with chronic diseases. This study integrates the UTAUT model with the Health Belief Model (HBM) to analyze key factors influencing the adoption of wearable devices by patients with chronic diseases, aiming to provide a more comprehensive understanding of their behavioral patterns and motivations.

**Methodology:**

A cross-sectional survey was conducted among Chinese patients with chronic diseases, yielding 432 valid responses. Partial Least Squares Structural Equation Modeling (PLS-SEM) was employed to construct the analytical model and examine the effects of latent variables on patients’ behavioral intention and actual use of wearable devices.

**Findings:**

The findings reveal that performance expectancy, effort expectancy, social influence, and facilitating conditions have a significant, positive influence on behavioral intention, which, in turn, positively affects actual use behavior. Performance expectancy mediates the relationships between social influence, perceived susceptibility, and perceived severity on behavioral intention. However, physical activity does not moderate the relationship between Performance Expectancy and Behavioral Intention.

**Conclusion:**

Performance expectancy, effort expectancy, social influence, and facilitating conditions are identified as key determinants of patients with chronic diseases’ adoption intention. Additionally, patients’ perceived severity and perceived susceptibility indirectly influence their usage intention through performance expectancy.

**Implications:**

These findings provide a theoretical foundation and practical guidance for optimizing the use of wearable devices in the management of chronic diseases. The study suggests that product development should focus on enhancing device performance, simplifying operational procedures, and strengthening social support systems.

## 1 Introduction

Non-communicable Diseases (NCDs) are characterized by their gradual onset, prolonged duration, recurrent nature, and often limited treatment efficacy, with some conditions being essentially incurable. These diseases have emerged as the predominant global health burden in contemporary healthcare. According to the World Health Organization’s (WHO) “World Health Statistics Report 2025,” In 2021, approximately 18 million people under the age of 70 years died from an NCDs globally, accounting for over half of all deaths in that age group, with cardiovascular diseases, cancer, chronic respiratory diseases, or diabetes showing particularly high mortality rates (1). In China, there has been a concerning trend of NCDs affecting increasingly younger populations, with NCD-related deaths accounting for over 80% of total mortality (2).

Progressive development and recurrent clinical manifestations necessitate long-term, sustained health management strategies, a characteristic of chronic diseases. With the rapid advancement of digital health technologies, wearable devices have emerged as innovative tools for health monitoring, playing a crucial role in chronic disease management. Wearable devices, defined as sensor-equipped apparatus designed for human use (3, 4), have been extensively implemented in monitoring and managing various chronic conditions, including hypertension (5), cardiac diseases (CD) (6), diabetes mellitus (DM) (7), chronic obstructive pulmonary disease (COPD) (8), and cancer.

These devices facilitate continuous monitoring and early diagnosis, providing scientific evidence for personalized treatment and effective management of chronic diseases. Furthermore, the onset and progression of chronic diseases are closely linked to physical activity levels, with physical inactivity representing a significant, modifiable risk factor. Regular exercise has been shown to reduce the risk of chronic diseases substantially (9).

Wearable devices, through the integration of multiple biosensors, enable precise monitoring of physical activity parameters (such as step count, energy expenditure, and exercise intensity) and chronic disease-related physiological indicators (including heart rate, blood pressure, and blood oxygen saturation) (10–12). These capabilities provide crucial data support for chronic disease management, with real-time monitoring systems showing particular promise for early detection and prevention.

Despite the flourishing market for wearable devices and the continuous emergence of innovative products, such as smartwatches and health monitors, patients with chronic diseases, a population with specific needs, must consider multiple factors when deciding whether to adopt these technologies. Previous research has predominantly employed the Technology Acceptance Model (TAM) (13) or its extended version, the Unified Theory of Acceptance and Use of Technology (UTAUT) (14, 15), to analyze factors influencing wearable technology adoption among the general population (see Table 1).

**Table 1:** Summary of related literature.

Author (year)	Objective/context	Methodology	Theory	Results/findings
Chuah et al. (21)	Examine drivers of smartwatch adoption intention among non-users; assess whether smartwatches are perceived as technology, fashion accessory, or both “fashnology”	Quantitative cross-sectional survey of Malaysian business students (*n* = 226); 7-point Likert scales; SEM for hypothesis testing; *post hoc* multi-group style analysis linking fashion vs. technology perceptions to antecedents	TAM extended with visibility construct and fashion/technology categorization	Perceived usefulness and visibility showed positive influence on attitude toward using smartwatches, while attitude demonstrated a positive impact on adoption intention. While perceived ease of use had an indirect effect on attitude through perceived usefulness, it also showed a strong relationship with perceived usefulness. Visibility was found to directly affect adoption intention. Regarding perception, consumers predominantly viewed smartwatches as technology (*m* = 5.61) rather than fashion accessories (*m* = 4.88).
Lee and Lee (3)	To examine factors that influence an individual’s intention to adopt a wearable fitness tracker, focusing on comparing two groups: those who were aware vs. unaware of fitness trackers	Quantitative – Survey with ordered logistic regression; Sample of 616 respondents (including both aware and unaware groups)	Interpersonal influence theory, Self-efficacy theory, and Consumer innovativeness theory	The study revealed significant differences in adoption intentions between groups aware and unaware of wearable fitness trackers, with stronger adoption intentions among those who were already aware of the devices. The analysis identified three key factors that showed statistically significant and positive associations with adoption intention across both groups: consumer attitudes, personal innovativeness, and health interests. This suggests that regardless of prior awareness, these three elements consistently influence an individual’s intention to adopt wearable fitness trackers.
Yildirim and Ali-Eldin (18)	To analyze factors influencing employees’ intention to use wearable devices at the workplace	Quantitative – Survey with Partial Least Square (PLS) path modeling and Adaptive Neuro-Fuzzy Inference System (ANFIS); Sample: 76 employees from an IT consulting firm	TAM, Theory of Reasoned Action (TRA), Theory of Planned Behavior	The study revealed that perceived usefulness emerged as the strongest motivating factor for individuals to adopt wearable IoT devices in the workplace. The analysis demonstrated that applying the ANFIS approach enhanced the predictability of user intention to use IoT devices. The demographic analysis showed that the majority of respondents (88%) were male, with 37% falling in the 45–54 age range and 25% in the 25–34 age range.
Wang et al. (35)	To develop and empirically test an integrated model combining UTAUT and TTF to understand consumer acceptance of healthcare wearable devices (HWDs)	Quantitative – Survey with Partial Least Squares Structural Equation Modeling (PLS-SEM); Sample: 406 valid responses	Unified Theory of Acceptance and Usage of Technology (UTAUT) and Task-Technology Fit (TTF) models	The study found that both task characteristics and technology characteristics served as significant determinants of task-technology fit. The analysis revealed important mediating relationships, with task-technology fit and effort expectancy acting as mediating variables between technology characteristics and behavioral intention. This suggests that the technical features of HWDs influence user acceptance both directly and indirectly through their impact on perceived ease of use and task-technology alignment.
Mattison et al. (27)	Examine the influence of wearables on health care outcomes in individuals with chronic diseases	A narrative systematic review was conducted by searching six databases for studies published between January 2016 and July 2021. Data extraction was based on six key themes, and risk of bias was assessed using the Cochrane Collaboration’s tool for RCTs and ROBINS-I for non-randomized studies.	Systematic review	The most studied conditions were type 2 diabetes (13%), Parkinson’s disease (10%), and chronic lower back pain (10%). Among 155 total outcome measures analyzed, the vast majority (89.7%) focused on healthcare outcomes, including pain (7.5%), quality of life (4.8%), and physical function (3.4%). Patient experience represented 7.7% of outcomes, while clinician experience and cost each accounted for only 1.3%. Notably, 3D virtual reality systems demonstrated positive effects on chronic pain, though results were mixed regarding exercise capacity, weight, and disease biomarkers.
Chen et al. (15)	To explore factors influencing elderly users’ intentions to accept wearable devices and develop a suitable theoretical framework for analyzing their acceptance	Quantitative – Survey, PLS-SEM	The study employed an improved version of the UTAUT model. The original UTAUT model was modified by incorporating new parameters specific to both wearable devices and elderly users.	The findings revealed that multiple device-related factors significantly influenced elderly users’ intention to use wearable devices, including performance expectancy, perceived cost, hedonic value, and aesthetic appeal. Regarding actual usage behavior, three individual factors showed significant impact: personal innovativeness in information technology, personal physiological condition, and intention to use. Notably, the study found no significant relationship between health anxiety and actual usage behavior.
Wu and Lim (32)	To identify and explore key factors influencing older adults’ willingness to adopt smart wearable devices and their impact mechanisms	Empirical analysis through a questionnaire survey of 389 older adult respondents to validate the model’s applicability and explore key influencing factors	The study integrated two theoretical frameworks: UTAUT2 and the Technology Readiness Index theory. The UTAUT2 model contributed factors such as performance expectancy, effort expectancy, social influence, facilitating conditions, hedonic motivation, and price value, while the TRI theory added personality trait dimensions including optimism, innovativeness, discomfort, and insecurity.	Digital health literacy emerged as the strongest direct positive influence, followed by price value, hedonic motivation, effort expectancy, performance expectancy, social influence, and facilitating conditions. Regarding personality traits, innovativeness and optimism positively influenced performance expectancy, while discomfort showed a negative impact. For effort expectancy, optimism had the strongest positive influence, followed by innovativeness, while both discomfort and insecurity showed negative effects.
Bertolazzi et al. (29)	To conduct an integrative systematic review examining factors that facilitate or hinder health technology adoption by older adults with chronic diseases	Integrative systematic review of research evidence from four electronic databases (PsycArticles, Scopus, Web of Science, and PubMed). Quality assessment using Mixed Methods Appraisal Tool (MMAT).	Not explicitly stated; used integrative systematic review methodology to synthesize and analyze existing research	The analysis identified five crucial domains that influence technology acceptance: demographic and socioeconomic factors, health-related factors, dispositional factors, technology-related factors, and social factors. The majority of the examined studies concentrated on individuals with comorbid conditions, with telemedicine tools emerging as the predominant technological intervention.
Tanaka et al. (71)	To explore the perspectives of Japanese elders, their caretakers, and healthcare providers on the use of technology and wearable devices for health monitoring at home	Qualitative study using a grounded theory approach	The study employed grounded theory methodology, which is an inductive approach that allows researchers to develop theoretical explanations from data collected in the field.	Healthcare workers specifically noted the value of monitoring technologies during COVID-19 isolation periods. However, several barriers to adoption were identified, primarily related to cost and technical complexity. Despite these challenges, the study indicated strong potential for wearable technology in supporting Japan’s aging population, though participants emphasized the need for simple, affordable solutions. Healthcare providers viewed the technology as particularly promising for remote monitoring and continuous health assessment.
Alzghaibi (7)	To investigate perceptions of people with diabetes regarding wearable devices, emphasizing perceived advantages, challenges, and potential role in facilitating diabetes self-management	The study employed a cross-sectional research design involving 418 people with diabetes. Data collection was conducted using a structured questionnaire that incorporated multiple data collection methods: Likert-scale items, multiple-choice questions, and open-ended responses.	The study’s approach to examining user perceptions, barriers, and adoption intentions aligns with established models of healthcare technology acceptance. The investigation of perceived benefits and challenges reflects theoretical constructs common to behavioral change theories in healthcare settings.	83.9% of participants recognizing their utility in monitoring glucose levels and physical activity. Participants reported significant benefits, including increased medication adherence (81.9%) and improved confidence in diabetes management (82.1%). However, several key barriers to adoption were identified, with data privacy concerns being the most prevalent (79.7%), followed by cost issues (77.0%) and usability challenges (75.1%). Thematic analysis of open-ended responses highlighted participants’ desire for specific features, including actionable feedback, seamless integration with healthcare providers, and enhanced usability. Notably, 81.9% of participants expressed willingness to adopt AI-integrated wearable devices if recommended by their healthcare providers, suggesting strong potential for widespread adoption despite the identified challenges.
Chandrasekaran et al. (24)	To explore usage and data-sharing practices of wearable devices among US adults during the later phases of the COVID-19 pandemic, examining both willingness to share wearable data and actual data-sharing behavior	Cross-sectional study using the National Cancer Institute’s Health Information National Trends Survey 6 (March–November 2022) with 5,591 US adult respondents.	The study appears to be grounded in technology adoption and health behavior frameworks, examining factors influencing usage and data sharing practices in healthcare settings.	The study documented a notable increase in wearable device adoption to 36.36% in 2022, up from 28 to 30% in 2019, while revealing a substantial gap between willingness to share data with healthcare providers (78.4%) and actual sharing behavior (26.5%). Analysis showed higher adoption rates among females (OR 1.49) and those with higher income levels (OR 2.65–3.2), while age was negatively correlated with both usage and data sharing. Hispanic respondents demonstrated higher willingness but lower actual sharing compared to African American respondents. The frequency of provider visits (OR 1.23) and total medical conditions (OR 1.35) emerged as significant predictors of data-sharing behavior.

However, the applicability of these models to patients with chronic diseases requires further validation. Notably, while UTAUT theory primarily focuses on technology-related factors, the adoption of wearable devices for health monitoring by patients with chronic diseases represents not merely a technological adoption behavior but also a health management practice. Consequently, relying solely on the UTAUT model may not fully elucidate the decision-making mechanisms underlying patients with chronic diseases’ adoption of wearable devices.

The Health Belief Model (HBM) emphasizes factors such as individuals’ perceptions of health threats, which play crucial roles in health management. Additionally, Research indicates that wearable devices can promote physical activity levels (16, 17). However, the relationship between the physical activity of patients with chronic disease and their willingness to adopt wearable devices remains underexplored. This involves not only technology acceptance issues but also closely relates to the health behavior patterns of chronic disease patients, warranting further investigation.

This Research covers the following research gaps. It explores the impacts of performance expectancy (PEX), effort expectancy (EEX), social influence (SI), perceived susceptibility (PSU), and perceived severity (PSE) on behavioral intention (BI). Secondly, it examines the impacts of PSU, PSE, and SI on PEX. Furthermore, it explores the impact of facilitating conditions (FC) on BI. Additionally, it measures the impact of BI on actual use. Lastly, this Research measures the indirect moderating impact of physical activity on the relationship between PEX and BI. This research is based on the UTAUT model and integrates the HBM to analyze key factors influencing the use of wearable devices by chronic disease patients. It aims to reveal the decision-making mechanisms underlying patients with chronic diseases’ adoption of wearable technology, thereby providing insights to improve health management and prevent disease progression through wearable devices.

This study makes several important theoretical and practical contributions to the field of digital health technology adoption. First, while existing research has predominantly examined wearable device adoption among general populations using technology acceptance frameworks, limited attention has been paid to chronic disease patients—a population with distinct health management needs and decision-making patterns. This study addresses this critical gap by explicitly focusing on chronic disease patients’ adoption behaviors, providing targeted insights for this vulnerable yet underexplored demographic. Second, this research advances theoretical understanding by integrating the UTAUT model with the HBM, bridging technology acceptance theory with health behavior theory. This integration acknowledges that wearable device adoption among chronic disease patients represents not merely a technological decision but also a health management behavior influenced by perceived health threats and disease severity. Third, this study innovatively examines the mediating role of PEX in the relationship between health beliefs (PSU and PSE) and BI, revealing the psychological mechanisms through which health threat perceptions translate into technology adoption. Fourth, by investigating physical activity as a potential moderator, this research explores the interplay between patients’ existing health behaviors and their technology adoption intentions, offering new insights into individual differences. From a practical perspective, the findings offer evidence-based guidance for healthcare providers, device manufacturers, and policymakers in designing targeted interventions, optimizing device features, and developing effective promotional strategies to enhance wearable device adoption among chronic disease patients. Ultimately, this research contributes to improving chronic disease management outcomes and reducing the global healthcare burden through enhanced technology-enabled self-management.

The remainder of this paper is structured as follows. Section 2 reviews relevant literature on wearable devices and establishes the theoretical foundation by integrating the UTAUT model with the HBM, culminating in the development of research hypotheses. Section 3 outlines the research methodology, including the research model, data collection procedures, sample characteristics, and analytical approach. Section 4 presents the empirical results, including measurement model assessment and hypothesis testing using PLS-SEM. Section 5 discusses the findings in relation to existing literature and their theoretical and practical implications. Finally, Section 6 concludes with a summary of key contributions, limitations, and directions for future research.

## 2 Literature review and theoretical foundation

### 2.1 Usage of wearable devices

Wearable devices are electronic devices that can be worn on the body or integrated into clothing or other bodily accessories. These devices utilize sensors and sensing technologies to collect physiological and environmental data, which is then transmitted to terminal devices for processing and analysis (18, 19). Such devices encompass smartwatches, smart bands, and smart patches, among which smartwatch-type devices have emerged as the most widely adopted wearable technology in clinical applications due to their multifunctional integration capabilities and user-friendly interfaces (20).

Research on wearable devices has primarily focused on several key dimensions: device cost-effectiveness and performance metrics (21, 22), as well as data security considerations (23, 24).

Wearable devices, as emerging technologies, have gained widespread adoption in personal health management and healthcare. Health monitoring is the fundamental functionality of these devices, which use integrated sensors to collect users’ physiological data in real time. Research has demonstrated that wearable devices can reliably track variations in fatigue and inflammatory responses (25) and predict cardiovascular and metabolic health outcomes (20, 26). These capabilities not only enable users to gain insights into their health status but also provide healthcare professionals with valuable reference information for their care.

Beyond physiological health monitoring, wearable devices have increasingly expanded their scope to encompass psychological health. The social functionality embedded in wearable devices has demonstrated positive implications for managing psychological health. Users can leverage these devices to share health-related data with their social connections, thereby strengthening social bonds and enhancing psychological well-being (24) (see Table 1).

For individuals with chronic conditions, the application of wearable devices holds particular significance (27) (see Table 1). Through continuous monitoring and data recording, patients can achieve early disease diagnosis and enhanced management of their health conditions. Research has demonstrated that wearable device data has successfully detected risks associated with Type 2 diabetes, hypertension, and cardiovascular diseases (22). Technological interventions, including wearable devices, have facilitated increased physical activity among users, thereby reducing disease risks in patients with chronic conditions (28, 29). This self-management approach has not only improved patients’ quality of life but also reduced the healthcare burden (3, 25).

### 2.2 Rationale for integrating UTAUT and HBM

The integration of UTAUT and HBM in this study addresses the multidimensional nature of wearable device adoption among chronic disease patients. UTAUT, as a technology acceptance model, effectively explains adoption behavior through technological and social factors, including PEX, EEX, SI, and facilitating conditions (30). However, UTAUT alone may not fully capture the health-specific motivations that drive chronic disease patients’ adoption decisions (31). HBM complements UTAUT by incorporating health belief constructs—specifically PSU and PSE—that reflect individuals’ perceptions of health threats and disease consequences. For patients with chronic diseases, wearable device adoption represents both a technology acceptance decision and a health management behavior. These patients evaluate devices not only based on technological utility but also on their potential to mitigate health risks and manage disease progression. By bridging these two theoretical perspectives, this study’s integrated model captures the interplay between technology-oriented factors and health-oriented beliefs, providing a more comprehensive framework for understanding adoption intentions. Furthermore, this integration allows us to examine how health beliefs may influence technology perceptions, specifically whether perceived health threats enhance the perceived performance value of wearable devices, thereby offering more profound insights into the psychological mechanisms underlying adoption behavior.

### 2.3 Theoretical foundation and research hypotheses

#### 2.3.1 UTAUT model

The UTAUT was developed as an extension of previous frameworks, including the TAM, to elucidate the mechanisms and factors influencing the acceptance and utilization of new technologies, including healthcare-related technologies (14). The model proposes four core constructs: PEX, EEX, SI, and FC, which have been extensively employed in Research examining BI and actual usage of new technologies.

BI represents an individual’s overall psychological disposition toward using a specific service or system, reflecting their subjective likelihood of adoption. PEX refers to the degree to which an individual believes that using new technology will yield positive effects on their performance enhancement. In the context of wearable devices, PEX manifests as users’ perceptions of how effectively these devices can optimize their health management. Empirical studies have consistently identified PEX as the strongest predictor of BI (14, 32). In the healthcare domain, PEX has been validated as the most crucial determinant influencing users’ continued usage intention (33). Patients with chronic diseases are more likely to adopt wearable devices if they perceive these devices as effective in improving their healthcare outcomes. Therefore, we hypothesize:

*H1*: PEX positively influences chronic disease patients’ BI to adopt wearable devices.

The EEX is defined as the degree of ease with which users use a system or technology (14). Previous Research has demonstrated a positive correlation between EEX and technology acceptance (34, 35), indicating that users exhibit significantly higher acceptance and usage intention when they perceive a technology as requiring minimal effort to use. In chronic disease management, when patients find wearable devices easy to operate and use, they are not only more likely to expect these devices to support their health management effectively but also more inclined to continue using them. Therefore, we hypothesize:

*H2*: EEX positively influences chronic disease patients' BI to adopt wearable devices.

The SI is defined in technology acceptance theory as the degree to which an individual perceives that important others (such as family members, friends, and doctors) expect them to adopt a particular technology or system (14). It has been established as a crucial antecedent of technology acceptance behavior (24, 36). Patients with chronic diseases, who often face high uncertainty and information asymmetry in their health management process, are particularly susceptible to external social factors influencing their decision-making. When making health-related decisions, patients typically reference and incorporate opinions and suggestions from important others. This SI can enhance patients’ willingness to use and accept wearable technology. Research has confirmed a positive relationship between SI and users’ BI to use health wearable devices (35). However, the role of SI in technology acceptance decisions is complex, primarily operating through three mechanisms: compliance, internalization, and identification (37). Additionally, positive SI has been found to correlate positively with users’ PEX of technology (38). When patients with chronic diseases are influenced by healthcare professionals, friends, or family members, they tend to develop higher performance expectations for wearable devices, which consequently increases their willingness to use them. Therefore, we hypothesize:

*H3*: SI positively influences chronic disease patients’ BI to adopt wearable devices.

*H4*: SI is positively related to the PEX of wearable devices among chronic disease patients.

*H5*: SI positively influences chronic disease patients' BI to adopt wearable devices through PEX.

The FC refers to the degree to which an individual perceives that organizational and technical infrastructure exists to support the use of a system. In the healthcare context, FC primarily encompasses the knowledge, skills, and support required to operate wearable devices, including connecting them to backend systems and synchronizing data to cloud servers. Previous Research has demonstrated a direct association between FC and BI (39), showing that FC enhances users’ BI to accept the technology (32, 34). When patients with chronic diseases possess the necessary knowledge and technical skills to operate wearable devices or have access to relevant support, their likelihood of adopting these devices increases. Therefore, we hypothesize:

*H6*: FC positively influences chronic disease patients' BI to adopt wearable devices.

The BI has been established as one of the most effective predictors of actual behavior. Related Research has demonstrated that BI positively influences the use of mobile health services (40). For patients with chronic diseases, a stronger intention to use wearable devices is associated with a higher likelihood of adopting and using the technology. Therefore, we hypothesize:

*H7*: Chronic disease patients' BI to use wearable devices positively influences their actual usage behavior.

#### 2.3.2 Health belief model

The HBM is one of the earliest classic theoretical frameworks for predicting individual health behaviors. This theory emphasizes the explanation of health behaviors in terms of individual attitudes and beliefs (41). According to Becker et al. (42), factors influencing individuals’ adoption of specific health-related behaviors primarily depend on two aspects: threat appraisal and outcome expectations. Threat appraisal encompasses individuals’ perceptions of disease susceptibility (PSU) and severity (PSE), while outcome expectations involve individuals’ assessment of potential benefits derived from adopting health behaviors. As people’s awareness of health threats continues to increase, there is a growing tendency to adopt smart devices for chronic disease management and disease prevention (43). Smart devices effectively enhance individuals’ perception of health threats and promote positive health behaviors through real-time physiological monitoring, health feedback, and personalized recommendations.

The PSU represents an individual’s subjective perception of potential threats and constitutes a health risk belief. When individuals perceive themselves as highly susceptible, they are more likely to adopt preventive behavioral measures (44). Research on patients with diabetes has demonstrated a positive correlation between PSU and the intention to use digitally delivered diabetes prevention programs (45). When patients with chronic diseases perceive themselves as susceptible to health problems, they tend to have higher PEX toward wearable devices and are more likely to adopt these devices for convenient access to health information, health management, and disease prevention. Therefore, we hypothesize:

*H8*: Chronic disease patients' PSU positively influences their BI to adopt wearable devices.

*H9*: Chronic disease patients' PSU positively influences their PEX toward wearable devices.

*H10*: Chronic disease patients' PSU positively influences their BI to adopt wearable devices through PEX.

The PSE refers to an individual’s subjective assessment and perception of the seriousness of a disease or health risk (46). As a foundation for help-seeking and self-directed behavior, higher perceived disease severity is associated with greater health-related pressure, thereby promoting more proactive health protection behaviors (47). Previous Research has demonstrated that individuals with higher levels of perceived threat typically exhibit stronger health motivation, higher PEX toward health technologies, and greater willingness to adopt such technologies (48). Similarly, when patients with chronic diseases maintain high awareness of the potential consequences of PSE and hold positive attitudes toward the PEX of wearable devices, their adoption intention increases significantly. Therefore, we hypothesize:

*H11*: The PSE of chronic disease patients positively influences their intention to adopt wearable devices.

*H12*: The PSE of patients with chronic diseases has a positive influence on their PEX toward wearable devices.

*H13*: The PSE of chronic disease patients positively influences their BI in adopting wearable devices through PEX.

#### 2.3.3 Wearable devices and physical activity (PA)

Physical activity refers to any bodily movement produced by skeletal muscles that requires energy expenditure. For patients with chronic diseases who face elevated health risks, physical activity is often considered a crucial preventive measure. Wearable devices can accurately track physical activity metrics, including step count, energy expenditure, sleep duration, and time spent at various activity levels (7, 49). Research has demonstrated the significant effectiveness of wearable devices in enhancing physical activity levels (17, 50, 51). Walking, as one of the most common forms of physical activity, is particularly effective for patients with diabetes when monitored with pedometers (9) and can reduce the risk of cardiovascular events among patients with chronic diseases (52, 53). Studies have also shown that wearable activity trackers and pedometers can serve as practical tools for improving health outcomes among cancer patients (17). Conversely, individuals’ physical activity levels indirectly influence their intention to adopt wearable devices (54). Patients with chronic diseases who engage in higher levels of physical activity may be more likely to use wearable devices for continuous monitoring of exercise data than those with lower activity levels. Therefore, we hypothesize:

*H14*: The physical activity level of chronic disease patients moderates the relationship between PEX of wearable devices and BI to use wearable devices.

## 3 Materials and methods

### 3.1 Research design

This study employed a cross-sectional survey design to investigate the determinants of wearable device adoption among patients with chronic diseases. The research employed an integrated theoretical framework combining UTAUT and HBM. A mixed-mode survey approach was adopted, combining both online and offline data collection methods to maximize response rates and ensure sample representativeness.

### 3.2 Participants and sampling

The study sample comprised adult patients with clinically confirmed chronic diseases who received their diagnosis, treatment, and follow-up care in hospital settings. The Research employed a mixed-mode survey approach, combining both online and offline methods. A total of 550 questionnaires were distributed across five hospitals, both on-site and via online channels, including WeChat and telephone interviews. Of these, 520 questionnaires were returned, of which 432 were deemed valid after incomplete responses were removed, yielding an effective response rate of 78.55%.

Prior to data collection, ethical approval was obtained from the relevant institutional review board, and informed consent was secured from all participants. Participants were assured of the confidentiality and anonymity of their responses. The questionnaire took approximately 15–20 min to complete.

To ensure data reliability and research relevance, strict inclusion criteria were established as follows: (1) age ≥18 years; (2) diagnosis of chronic conditions (such as hypertension, diabetes, chronic obstructive pulmonary disease, etc.) confirmed by medical institutions; and (3) active use of wearable technology devices (e.g., smartwatches) for health monitoring during the study period.

Based on the analysis of valid samples, Table 2 shows that among patients with chronic diseases using wearable devices, 232 (53.7%) were male and 200 (46.3%) were female. The majority of patients (63.6%) were aged 21–40 years. A substantial proportion (71.1%, *n* = 307) held bachelor’s degrees or higher.

**Table 2:** Basic information of interviewees.

Sample	Category	Number	Percentage (%)
Gender	Male	232	53.7
	Female	200	46.3
Age	18–20	18	4.2
	21–30	160	37.0
	31–40	115	26.6
	41–50	80	18.5
	51–60	33	7.6
	61–70	15	3.5
	≥70	11	2.5
Education level	Junior high school or below	26	6.0
	High school/technical school	32	7.4
	College diploma	67	15.5
	Bachelor’s degree	234	54.2
	Graduate degree	73	16.9
Chronic conditions	Hypertension	124	28.7
	Coronary heart disease	27	6.3
	Hyperlipidemia	36	8.3
	Cerebral hemorrhage	8	1.9
	Chronic heart failure	6	1.4
	Rheumatic heart disease	5	1.2
	Chronic obstructive pulmonary disease	19	4.4
	Chronic bronchitis	21	4.9
	Gout	48	11.1
	Obesity	86	19.9
	Diabetes	43	10.0
	Osteoporosis	16	3.7
	Chronic gastritis	35	8.1
	Chronic hepatitis	16	3.7
	Chronic nephritis	32	7.4
	Osteoarthritis	58	13.4
	Cancer	20	4.6
	Neurological disorders	18	4.2
	Other chronic diseases	33	7.6
Disease duration	<1 year	59	13.7
	1–5 years	270	62.5
	5–10 years	76	17.6
	11–15 years	16	3.7
	≥16 years	11	2.6
Wearable device type	Huawei smartwatch	242	56.0
	Apple Watch	63	14.6
	Samsung smartwatch	10	2.3
	Xiaomi smartwatch	109	25.2
	Fitbit series	3	0.7
	Smart insoles	4	0.9
	Smart glasses	7	1.6
	Others	7	1.6
Duration of wearable device usage	Less than 1 year	124	28.7
	1–3 years	222	51.4
	3–5 years	55	12.7
	More than 5 years	31	7.2
Physical activity levels	Level 1	168	38.9
	Level 2	232	53.7
	Level 3	32	7.4

Regarding disease duration, 59 patients (13.7%) had been diagnosed within the past year, 270 patients (62.5%) had lived with their condition for 1–5 years, and 76 patients (17.6%) had lived with their condition for 6–10 years.

**Figure 1:**
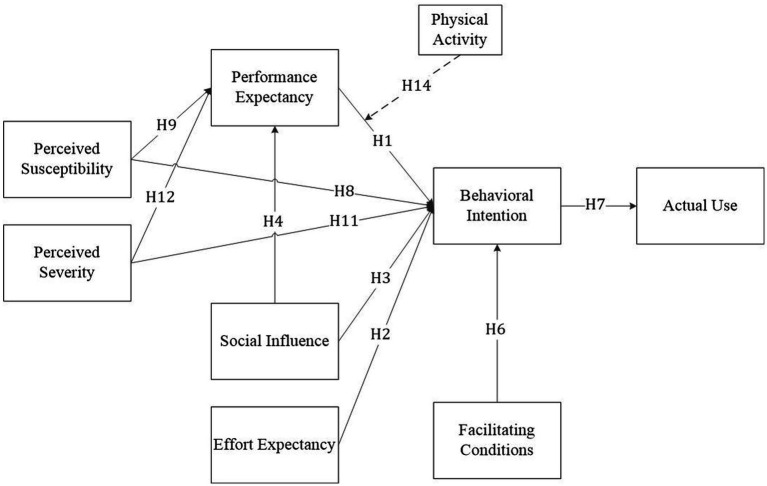
Theoretical model. Solid line = direct impact; dotted line = indirect impact.

Regarding wearable devices (clinically approved monitoring equipment or devices priced at 1000 RMB or above), smart watches were the predominant choice. Huawei watches were used by 242 patients (56.0%), followed by Xiaomi smart watches (109 patients, 25.2%), Apple watches (63 patients, 14.6%). A small number of patients used other devices, such as Fitbit devices, smart insoles, and smart glasses. The theoretical frame work of the study is indicated in Figure 1.

The assessment of physical activity levels among patients with chronic diseases revealed that 168 patients (38.9%) maintained low physical activity levels, 232 (53.7%) exhibited moderate physical activity levels, and only 32 patients (7.4%) achieved high physical activity levels.

### 3.3 Measures

The measurement instrument for this study, derived from previous Research, comprised 30 items measuring eight latent constructs: PSE (5 items), PSU (4 items), PEX (4 items), SI (3 items), EEX (3 items), FC (3 items), BI (4 items), and use behavior (4 items).

The scales for PSE and PSU were adapted from Champion (46) and Zhao et al. (55), with modifications to suit the current research context. The scales for PEX, EEX, SI, FC, and BI were modified from Venkatesh et al. (39) and Wang et al. (35). The use behavior scale was adapted from Alam et al. (40). Additionally, the physical activity scale, consisting of 9 items, was adopted from Craig et al. (56). The detailed questionnaire is presented in Appendix 1.

### 3.4 Data analysis

The collected data were analyzed using Partial Least Squares Structural Equation Modeling (PLS-SEM) with SmartPLS 4 software (57). PLS-SEM was selected as the primary analytical technique due to its advantages in handling complex models with multiple constructs, its minimal requirements regarding sample size and data distribution, and its suitability for exploratory research (58). The data analysis procedure consisted of two main stages.

#### 3.4.1 Measurement model assessment

The reliability and validity of the measurement model were evaluated through several criteria: (1) internal consistency reliability using Cronbach’s alpha and composite reliability (CR); (2) convergent validity through average variance extracted (AVE); and (3) discriminant validity using the Fornell-Larcker criterion and Heterotrait-Monotrait (HTMT) ratio.

#### 3.4.2 Structural model assessment

The structural model was evaluated to test the proposed hypotheses. This included examining: (1) the path coefficients and their statistical significance using bootstrapping procedures with 5,000 resamples; (2) the coefficient of determination (*R*^2^) to assess the model’s explanatory power; (3) the effect size (*f*^2^) to determine the substantive impact of predictor constructs; and (4) the predictive relevance (*Q*^2^) using blindfolding procedures.

To test the mediating effects of PEX, the bootstrapping method was employed following the recommendations of Preacher and Hayes (59). For the moderating effect of physical activity on the relationship between PEX and BI, a product indicator approach was utilized.

Common method bias was assessed using Harman’s single-factor test and the full collinearity test to ensure the validity of the findings.

## 4 Results

### 4.1 Outer model assessment

The Partial Least Squares (PLS) method is a statistical analysis technique based on structural equation modeling (SEM) used to test and validate the proposed model and the hypothesized relationships among constructs. Following Fornell and Larcker (60) and Hair et al. (61), internal reliability was assessed using factor loadings, composite reliability, and Cronbach’s alpha, while convergent validity was evaluated using the AVE. As shown in Table 3, all item factor loadings exceeded the recommended threshold of 0.6, composite reliability values for each construct were above the recommended value of 0.7, and Cronbach’s *α* values were higher than the recommended threshold of 0.7. The AVE values for all constructs exceeded the recommended threshold of 0.5, indicating satisfactory levels of reliability and validity for all constructs. Additionally, the Variance Inflation Factor (VIF) was employed to assess multicollinearity in the model, with values recommended to be below 3 (62). The results revealed that all VIF values were below the recommended threshold, indicating no significant multicollinearity issues among variables and ensuring the reliability of the model results.

**Table 3:** Reliability and convergent validity.

Factor	Item	Mean	Standard deviation	Factor loading	Composite reliability	Cronbach’s alpha	Average variance extracted	Variance inflation factor
Perceived severity (PSE)	PSE1	5.762	1.198	0.837	0.912	0.880	0.676	2.151
	PSE2	5.556	1.139	0.815				2.047
	PSE3	5.799	1.117	0.833				2.152
	PSE4	5.711	1.156	0.823				2.054
	PSE5	5.512	1.188	0.802				1.901
Perceived susceptibility (PSU)	PSU1	5.278	1.348	0.860	0.900	0.853	0.693	2.222
	PSU2	5.056	1.405	0.798				1.780
	PSU3	5.634	1.229	0.843				1.849
	PSU4	5.266	1.354	0.828				1.992
Performance expectancy (PEX)	PEX1	5.650	1.087	0.855	0.910	0.869	0.718	2.080
	PEX2	5.764	1.091	0.878				2.512
	PEX3	5.440	1.161	0.828				1.993
	PEX4	5.852	1.032	0.826				1.966
Behavioral intention (BI)	BI1	5.870	1.030	0.871	0.924	0.891	0.753	2.419
	BI2	5.729	1.160	0.879				2.574
	BI3	5.759	1.121	0.863				2.344
	BI4	5.609	1.199	0.859				2.190
Effort expectancy (EEX)	EEX1	5.924	1.028	0.891	0.908	0.849	0.768	2.080
	EEX2	5.833	0.986	0.873				2.056
	EEX3	5.907	1.039	0.865				2.057
Social influence (SI)	SI1	5.433	1.260	0.892	0.916	0.863	0.784	2.188
	SI2	5.278	1.284	0.882				2.350
	SI3	5.368	1.235	0.882				2.119
Facilitating conditions (FC)	FC1	5.581	1.103	0.888	0.897	0.827	0.743	2.052
	FC2	5.623	1.134	0.876				2.065
	FC3	5.410	1.102	0.822				1.681
Actual use (USE)	USE1	5.898	1.067	0.849	0.899	0.851	0.691	2.076
	USE2	5.789	1.076	0.816				1.930
	USE3	5.655	1.040	0.816				1.881
	USE4	5.887	1.029	0.844				2.034

Discriminant validity refers to the degree to which constructs in the measurement model can effectively distinguish between different variables. Following the discriminant validity assessment criteria proposed by Fornell and Larcker (60), the square root of each construct’s AVE was greater than its correlations with other constructs, confirming discriminant validity (see Table 4). Additionally, this study employed the Heterotrait-Monotrait Ratio (HTMT) as a complementary method of testing. HTMT, which calculates the ratio between inter-construct correlations and internal consistency, provides a more sensitive measure of construct distinctiveness. All HTMT values in this study were below 0.85 (62), further supporting good discriminant validity.

**Table 4:** Discriminant validity.

Construct	FC	BI	EEX	PEX	PSE	PSU	SI	USE	FC	BI	EEX	PEX	PSE	PSU	SI
FC	**0.862**														
BI	0.514	**0.868**							0.596						
EEX	0.433	0.478	**0.876**						0.509	0.547					
PEX	0.460	0.606	0.405	**0.847**					0.544	0.687	0.466				
PSE	0.355	0.512	0.260	0.517	**0.822**				0.418	0.576	0.298	0.590			
PSU	0.373	0.461	0.332	0.494	0.497	**0.832**			0.442	0.522	0.379	0.568	0.574		
SI	0.435	0.518	0.288	0.523	0.454	0.376	**0.886**		0.512	0.586	0.332	0.597	0.520	0.436	
USE	0.476	0.530	0.421	0.440	0.339	0.332	0.431	**0.831**	0.564	0.606	0.488	0.508	0.390	0.385	0.499

FC, facilitating conditions; BI, behavioral intention; EEX, effort expectancy; PEX, performance expectancy; PSE, perceived severity; PSU, perceived susceptibility; SI, social influence; USE, actual use. Bold values represent the square root of each construct’s AVE.

Common Method Bias (CMB), a systematic error that may arise from single-source data collection or similar measurement methods, was also assessed. To evaluate CMB, Harman’s Single Factor Test was conducted. The results showed that the maximum variance explained by a single factor was 37.272%, which is below the critical threshold of 50% (62), suggesting that CMB was not a significant concern in this study.

### 4.2 Inner model assessment

In PLS-SEM, *R*^2^ Values are used to evaluate the model’s explanatory power, with higher values indicating greater explanatory power (63). As shown in Table 5, the six exogenous constructs (PSE, PSU, PEX, EEX, SI, and FC) explained 52.9% of the variance in wearable device use intention, while PSE and PSU accounted for 41.8% of the variance in PEX, both indicating moderate levels of explanation. However, the explanatory power of wearable device use intention on use behavior was relatively low at 28.1%.

**Table 5:** Direct empirical results.

No.	Path	Beta	Bias corrected (2.5, 97.5%)	*t*	*p*	*R^2^*	Q^2^	*f^2^*	Decision
H1	PEX → BI	0.252	(0.137, 0.363)	4.429	0.000	0.529	0.389	0.072	Supported
H2	EEX → BI	0.194	(0.110, 0.274)	4.560	0.000			0.059	Supported
H3	SI → BI	0.158	(0.054, 0.259)	3.033	0.002			0.034	Supported
H4	SI → PEX	0.315	(0.209, 0.418)	5.962	0.000	0.418	0.293	0.130	Supported
H6	FC → BI	0.163	(0.061, 0.260)	3.215	0.001			0.037	Supported
H7	BI→USE	0.530	(0.406, 0.638)	8.895	0.000	0.281	0.190	0.390	Supported
H8	PSU → BI	0.071	(−0.042, 0.191)	1.186	0.236			0.007	Rejected
H9	PSU → PEX	0.252	(0.154, 0.356)	4.902	0.000			0.079	Supported
H11	PSE → BI	0.172	(0.064, 0.286)	3.030	0.002			0.039	Supported
H12	PSE → PEX	0.249	(0.153, 0.352)	4.847	0.000			0.071	Supported

PEX, performance expectancy; EEX, effort expectancy; SI, social influence; FC, facilitating conditions; BI, behavioral intention; PSU, perceived susceptibility; PSE, perceived severity; USE, actual use.

**Figure 2:**
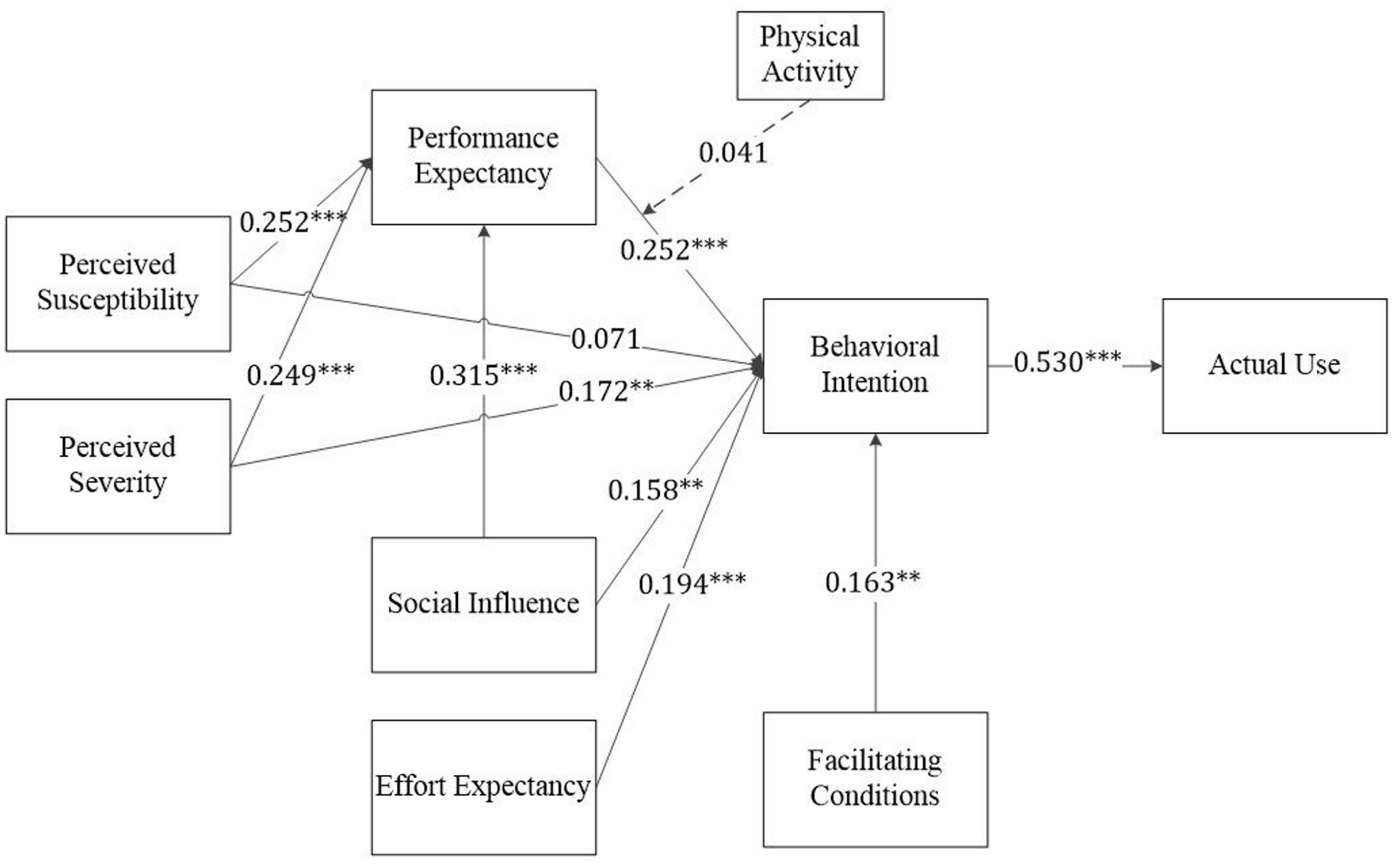
Empirical results. *** = *p* < 0.001, ** = *p* < 0.01.

We performed the blindfolding test to obtain *Q*^2^-values. As shown in Table 5 and Figure 2, the model’s predictive relevance (*Q*^2^) values were 0.190, 0.293, and 0.389, indicating small to medium predictive capability (61). Additionally, *f*^2^ values were used to assess the effect size between variables (63). The direct effects results are presented in Table 5. Within the UTAUT model, PEX (*β* = 0.252, *t* = 4.429, *p* < 0.001), EEX (*β* = 0.194, *t* = 4.560, *p* < 0.001), SI (*β* = 0.158, *t* = 3.033, *p* < 0.01), and FC (*β* = 0.163, *t* = 3.215, *p* < 0.01) all demonstrated significant positive effects on BI, supporting H1, H2, H3, and H6. PSE (*β* = 0.172, *t* = 3.030, *p* < 0.01) showed a positive effect on BI, while PSU (*β* = 0.071, *t* = 1.186, *p* > 0.05) showed no significant effect on BI. Thus, H11 was supported, but H8 was rejected. For chronic disease patients, SI (*β* = 0.315, *t* = 5.962, *p* < 0.001), PSU (*β* = 0.252, *t* = 4.902, *p* < 0.001), and PSE (*β* = 0.249, *t* = 4.847, *p* < 0.001) all demonstrated significant positive effects on PEX, supporting H4, H9, and H12. Furthermore, wearable device use intention positively affected use behavior (*β* = 0.530, *t* = 8.895, *p* < 0.001), supporting H7.

The mediation analysis results (see Table 6) confirmed that PEX partially mediated the relationships between SI and BI (*β* = 0.079, *t* = 3.573, *p* < 0.001) and between PSE and BI (*β* = 0.063, *t* = 3.180, *p* < 0.01). Additionally, PEX fully mediated the relationship between PSU and BI (*β* = 0.063, *t* = 3.150, *p* < 0.01). Therefore, H5, H10, and H13 were all supported.

**Table 6:** Indirect and moderation effects.

No.	Path	Beta	Bias corrected (2.5%, 97.5%)	*t*	*p*	Decision
H5	SI → PEX → BI	0.079	(0.042,0.131)	3.573	0.000	Supported
H10	PSU → PEX → BI	0.063	(0.031,0.112)	3.150	0.002	Supported
H13	PSE → PEX → BI	0.063	(0.030,0.110)	3.180	0.001	Supported
H14	PA x PEX → BI	0.041	(−0.036, 0.123)	1.001	0.317	Rejected

SI, social influence; PEX, performance expectancy; BI, behavioral intention; PSU, perceived susceptibility; PSE, perceived severity; PA, physical activity.

However, PA did not moderate the relationship between PEX and BI (*β* = 0.041, *t* = 1.001, *p* > 0.05), thereby rejecting H14.

## 5 Discussion

This study aimed to explore and validate the key factors influencing Chinese patients with chronic diseases’ willingness to use wearable devices. The cross-sectional analysis revealed that PEX, EEX, SI, and FC all positively influenced chronic patients’ usage intention, which, in turn, positively affected their actual usage behavior. PSE and PSU demonstrated both direct and indirect positive effects on patients’ adoption intention for chronic disease. Notably, integrating the UTAUT and HBM models provided a more effective explanation of patients’ intention to adopt wearable devices (*R*^2^ = 0.529).

The findings indicate that PEX emerged as the most critical predictor of patients with chronic diseases’ intention to use wearable devices (*β* = 0.252, *t* = 4.429, *p* < 0.001), consistent with previous studies by Song et al. (64), Yang and Koenigstorfer (65), and Wu et al. (32). Patients with chronic diseases generally demonstrated positive attitudes toward the health monitoring capabilities of wearable devices, acknowledging their practical value in monitoring vital physiological parameters. Their positive perceptions of device effectiveness significantly strengthened their intention to use the device. Qualitative interviews further corroborated these findings. For instance, a hypertensive patient noted: “By continuously wearing the device, I can compare changes in physiological indicators between normal and uncomfortable conditions. When experiencing symptoms like dizziness, headache, chest tightness, or nausea, the device’s blood pressure readings provide objective evidence for seeking timely medical attention.” Similarly, a diabetic patient reported: “The device data clearly shows significant blood glucose elevations when I consume fried foods or high-carbohydrate meals, which has motivated me to adjust my dietary habits actively.” Some patients verified the reliability of their devices by cross-referencing the readings with professional medical equipment at hospitals. This cross-validation not only enhanced patients’ trust in the devices but also optimized their disease self-management strategies, aligning with previous research findings (66). PEX serves as both a psychological mechanism driving patients with chronic diseases to adopt wearable technology and a key factor promoting its sustained use in long-term chronic disease management. These findings provide important theoretical foundations for optimizing the functionality of medical wearable devices.

Although patients with chronic diseases’ beliefs about wearable devices’ PEX are crucial, these beliefs are also influenced by device-related effort considerations. The study results demonstrate that EEX positively influences usage intention (*β* = 0.194, *t* = 4.560, *p* < 0.001), aligning with previous research (35, 64). The ease of use of wearable devices plays a pivotal role in the adoption of technology.

For chronic patients, if they only need to learn partial functionality of the device and can quickly master it, they are more likely to perceive the device as a suitable solution, consistent with related research findings (67). For example, when first using wearable devices such as smartwatches, although patients need to read instruction manuals and familiarize themselves with relevant functions, they can typically become proficient with relatively straightforward operations quickly. This perceived ease of use reduces the subjective effort required to use the technology, thereby increasing patients’ willingness to adopt it.

The study reveals that SI has a positive effect on both PEX (*β* = 0.315, *t* = 5.962, *p* < 0.001) and BI (*β* = 0.158, *t* = 3.033, *p* < 0.01) of wearable devices among chronic disease patients, consistent with previous findings (24, 68). SI shapes patients’ acceptance of wearable devices for chronic disease management through mechanisms such as social identification and social support.

When family members, friends, or healthcare professionals (doctors) endorse specific wearable devices, patients are more likely to try or continue using them. This support not only enhances patients’ trust in the devices but also increases their recognition of the devices’ value.

Furthermore, FC significantly influences the intention of chronic disease patients to adopt wearable devices (*β* = 0.163, *t* = 3.215, *p* < 0.01), aligning with previous research findings (69). People are more inclined to adopt new technologies when better conditions facilitate their use (32). The availability of adequate knowledge, technical support, and resources directly affects patients with chronic diseases’ acceptance and willingness to use wearable devices.

When patients with chronic diseases receive comprehensive knowledge and guidance, robust technical support, and necessary resource guarantees, both psychological barriers and operational thresholds to using technological products are significantly reduced. Establishing such support systems not only alleviates patients’ concerns about technological complexity but also enables them to focus on the practical health benefits of technological applications.

The findings demonstrate that patients’ health motivation positively influences their PEX of wearable devices, establishing a unique pathway linking HBM to PEX in UTAUT. PSE emerges as a significant predictor of patients’ intention to adopt wearable devices among those with chronic diseases (*β* = 0.172, *t* = 3.030, *p* < 0.01), consistent with previous research findings (44, 70, 71). Compared to healthy individuals, patients with chronic conditions typically have a clearer perception of their illness severity. When they perceive serious threats to their health status, their willingness to adopt wearable devices increases.

Further analysis reveals that PEX partially mediates the relationship between perceived threat and BI (*β* = 0.063, *t* = 3.180, *p* < 0.01), aligning with previous research findings (48). This indicates that the more severe the health threat perceived by patients, the higher their PEX of wearable devices, which, in turn, influences their ultimate BI.

Although this study finds that PSU has no direct effect on chronic disease patients’ intention to adopt wearable devices (*β* = 0.071, *t* = 1.186, *p* > 0.05), which contradicts some previous research findings (45), PSU indirectly influences BI through PEX, with PEX serving as a complete mediator between the two variables (*β* = 0.063, *t* = 3.150, *p* < 0.01).

In qualitative interviews, a hypertensive patient expressed, “Having lived with hypertension for 5 years, I live in constant fear, worried about severe consequences such as cerebral hemorrhage, myocardial infarction, and sudden syncope. The dynamic blood pressure monitoring function can alleviate this anxiety, making me willing to wear a smartwatch.” Due to their prolonged exposure to disease risks, chronic patients exhibit heightened sensitivity and anxiety toward health issues, leading them to develop higher expectations regarding the functionality and effectiveness of health monitoring devices. These expectations become a key driving force in their decision to adopt devices.

Physical activity has been recognized as a primary intervention for both primary and secondary disease prevention. Physical activity not only effectively prevents chronic diseases but also, in some instances, can delay disease progression, aid in treatment, improve quality of life, and potentially extend patients’ life expectancy (72).

However, this study’s findings indicate that the physical activity levels of chronic patients neither influence their intention to use wearable devices nor moderate the relationship between PEX and BI. This may be attributed to the fact that patients with chronic diseases, when observed without intervention, generally maintain low physical activity levels, with remarkably few patients engaging in high-intensity physical activities. Following diagnoses of chronic conditions such as hypertension or diabetes, most patients exhibit symptoms including poor sleep, reduced appetite, negative mood, and insufficient exercise, which may be related to psychological changes and medication effects.

Although patients’ physical activity levels do not directly affect their BI, interview results reveal that some patients with chronic diseases, through using wearable devices to monitor real-time data such as step counts and calorie expenditure, gain a better understanding of their physical condition. This monitoring capability motivates them to participate in physical activities actively and demonstrates increased enthusiasm and engagement in exercise.

## 6 Theoretical implications

This study integrates the HBM with the UTAUT to investigate key factors influencing the adoption of wearable devices by chronic disease patients, aiming to provide a more comprehensive explanation of their motivations and behaviors. This theoretical integration not only extends the applicability of both HBM and UTAUT but also offers a novel perspective on understanding technology acceptance behavior among patients with chronic diseases.

The findings reveal that PSE and PSU from HBM positively influence BI through PEX in the UTAUT model. This indicates that patients with chronic diseases’ perceptions of disease severity and susceptibility enhance their expectations for wearable technology’s role in disease management, thereby strengthening their BI and actual adoption behavior (*β* = 0.530, *t* = 8.895, *p* < 0.001). Chronic disease patients exhibit a unique dual cognitive processing mechanism, characterized by “threat assessment-technology assessment,” which has significant implications for designing precision intervention programs.

PEX in wearable devices is a crucial predictor of device adoption among patients with chronic diseases. Patients anticipate obtaining real-time health data and personalized recommendations through these devices to address their health challenges more effectively. This finding highlights the pivotal role of PEX within the TAM, particularly in healthcare management. Additionally, the perceived ease of use, FC, and SI positively affect the BI of chronic disease patients, collectively driving their technology acceptance behavior.

Although this study found that the physical activity levels of patients with chronic disease do not moderate the relationship between PEX and wearable device adoption intention, these devices demonstrate potential value in motivating patients’ physical activity participation and enhancing health awareness. Future Research should explore device functionality optimization and exercise intervention strategies to more effectively promote health management behaviors among patients with chronic diseases.

## 7 Practice implications

First, chronic disease patients’ health motivations, derived from PSU and PSE, are closely associated with their PEX of wearable devices. This relationship can enhance their recognition of wearable devices’ benefits and strengthen the advanced functions and medical monitoring applications of these devices. Chronic disease patients rely on wearable devices’ health-monitoring technology to enable dynamic tracking and prediction of their health status, regularly reporting data to healthcare providers. Based on these data, healthcare providers can develop more targeted treatment plans and recommendations, ensuring patients receive better monitoring and guidance. The practical utility of these devices- and, consequently, patients’ willingness to use them for personalized health management – would be significantly enhanced if wearable devices could not only monitor and assess conditions in real time but also alert users to abnormalities and even predict and warn of potential health issues before they manifest.

Second, the usability of wearable devices is a crucial factor, underscoring the importance of simple, user-friendly design to reduce EEX among patients with chronic diseases. These devices should feature clear, intuitive user interfaces that allow patients to easily navigate various functions, with enhanced interactive experiences such as gesture control and voice recognition for convenient operation. Given that many wearable devices need to connect with other functional devices across different operating systems, it is essential to address compatibility issues between these devices. This includes providing robust technical infrastructure and services to enable timely feature updates and bug fixes, as well as precise data synchronization and sharing, thereby simplifying the data collection process. Patients’ confidence in accessing professional technical support also represents a significant factor in adoption. Furthermore, during the promotion of health technology, emphasis should be placed on building positive social support networks. Support from healthcare professionals, family members, and friends can enhance users’ expectations of the technology’s performance, thereby promoting its adoption and continued use to achieve effective and sustainable chronic disease management.

Ultimately, implementing exercise interventions to increase physical activity among patients with chronic diseases gradually improves their health status and disease control. By leveraging multidimensional physiological data collected via wearable devices (such as heart rate variability, blood oxygen saturation, and exercise intensity) alongside patients’ clinical diagnostic results, individualized exercise prescriptions can be developed. These progressive exercise programs gradually increase the volume and intensity of physical activity, fostering sustainable exercise habits. Throughout this process, comprehensive consideration should be given to patients’ specific requirements for wearable devices to enable timely health monitoring and management.

## 8 Research limitations and future research directions

As a cross-sectional study, this Research has certain limitations in revealing the willingness and behavioral patterns of chronic disease patients regarding the use of wearable devices. First, the research design failed to capture the temporal dynamics of technology adoption. Future studies are recommended to employ longitudinal tracking designs with multi-point data collection to systematically examine the complete behavioral trajectories of chronic disease patients, from initial adoption to continued use or abandonment, and identify key influencing factors at each stage. Second, the physical activity levels of patients with chronic diseases in this study were assessed without intervention and were generally low. Future Research could incorporate evidence-based exercise intervention programs supported by objective monitoring data from wearable devices to more accurately assess the relationship between physical activity levels and device usage behavior.

## 9 Conclusion

This study contributes to the understanding of wearable device adoption among chronic disease patients by integrating the UTAUT model with the HBM. The findings reveal that PEX, EEX, SI, and facilitating conditions significantly influence BI, which in turn drives actual use behavior. Notably, PEX serves as a critical mediating mechanism through which health beliefs—specifically PSU and PSE—translate into adoption intentions, demonstrating a dual cognitive processing pathway. However, contrary to expectations, physical activity levels do not moderate the relationship between PEX and BI. These results underscore the importance of addressing both technological factors and health-related beliefs when promoting the adoption of wearable devices among chronic disease patients. From a practical perspective, healthcare providers and device manufacturers should focus on enhancing device performance, simplifying user interfaces, leveraging social support networks, and emphasizing health threat awareness to facilitate adoption. Future research should explore longitudinal adoption patterns, examine additional moderating factors, and investigate the effectiveness of targeted interventions across diverse chronic disease populations and cultural contexts.

## Data Availability

The raw data supporting the conclusions of this article will be made available by the authors, without undue reservation.
