# Supplementary material for: Bridging UTAUT and HBM: determinants of wearable device adoption among chronic disease patients
Source: Front Public Health. 2026 Jan 27;13:1687887. doi: 10.3389/fpubh.2025.1687887 (PMC12886379; doi:10.3389/fpubh.2025.1687887)
Supplement: Supplementary file 1 [file Supplementary_file_1.docx]

Appendix A

Constructs and Measurements

| Constructs | Measurement Items |
| --- | --- |
| Perceived Severity | My chronic disease affects my daily life, outdoor activities, and work |
|  | The treatment costs for chronic diseases are high. |
|  | Chronic disease causes a psychological burden. |
|  | Chronic disease brings a considerable burden and trouble to family members. |
|  | My chronic condition is a highly distressing disease. |
| Perceived Susceptibility | My current health status makes me susceptible to chronic diseases. |
|  | I frequently encounter health issues in daily life. |
|  | Lack of physical activity increases the likelihood of developing chronic diseases. |
|  | I may develop other diseases in the future. |
| Performance Expectancy | Using wearable devices helps manage and prevent chronic diseases. |
|  | Using wearable devices facilitates daily life. |
|  | Using wearable devices can help me increase my level of physical activity. |
|  | Using wearable devices helps me collect health information. |
| Effort Expectancy | I can use wearable devices proficiently with ease. |
|  | I find wearable devices easy to use |
|  | Learning to operate wearable devices is easy for me. |
| Social Influence | People important to me (e.g., family members) think I should use wearable devices. |
|  | People I value (e.g., healthcare providers) think I should use wearable devices. |
|  | People who influence my behavior (e.g., peer patients) think I should use wearable devices. |
| Facilitating Conditions | I have access to the necessary resources to utilize wearable devices. |
|  | I possess the necessary knowledge to operate the system. |
|  | Specific individuals (or groups) are available to help with system difficulties. |
| Behavioral Intention | I plan to utilize wearable devices to monitor my health status over the coming months. |
|  | I plan to use wearable devices frequently in the coming months. |
|  | I am willing to use wearable devices in the coming months. |
|  | I will recommend wearable devices to people around me. |
| Usage Behavior | I use wearable devices to record my physical data. |
|  | I use wearable devices to ensure health safety. |
|  | I use wearable devices regularly. |
|  | I use wearable devices continuously. |
